# Preoperative cardiovascular evaluation in patients with cancer

**DOI:** 10.3389/fcvm.2026.1780281

**Published:** 2026-06-12

**Authors:** Carl Zehner, Noah I. Beinart, Jung Hyun Kim, Andrea Ruesta Carrion, Angelica Paniagua-Bojorges, Silvia Fernanda López Moreno, Bernardo Casso-Chapa, Keila Ostos Mendoza, Venkata Subrahmanya Kumar Samanthapudi, Jonghae Lee, Kun Yuan Chu, Oanh Hoang, Gilbert F. Mejia, Anita Deswal, Nicolas L. Palaskas, Sivareddy Kotla, Nhat-Tu Le, Efstratios Koutroumpakis, Syed Wamique Yusuf, Jun-ichi Abe, Michael S. Ewer

**Affiliations:** 1 Department of Cardiology, The University of Texas MD Anderson Cancer Center, Houston, TX, United States; 2 Instituto Tecnológico y de Estudios Superiores de Monterrey, Escuela de Medicina y Ciencias de la Salud, Monterrey, Nuevo León, México; 3 Universidad Peruana Cayetano Heredia, Lima, Peru; 4 Department of Cardiovascular Sciences, Houston Methodist Research Institute, Houston, TX, United States

**Keywords:** cancer patients, cardio-oncology, multidisciplinary collaboration, preoperative evaluation, risk stratification, thromboprophylaxis

## Abstract

Cancer patients undergoing non-cardiac surgery represent a uniquely high-risk group for perioperative cardiovascular complications. These individuals frequently exhibit coronary artery disease, peripheral arterial disease, cardiomyopathy, valvular dysfunction, arrhythmias, pericardial disease, and heightened thrombotic or bleeding risk. Many surgeries in this population are time-sensitive rather than elective, limiting opportunities for optimization and requiring clinicians to balance competing risks. Current guidelines provide limited oncology-specific recommendations; however, several strategies can improve outcomes. These include incorporating cancer history into risk stratification, lowering thresholds for transthoracic echocardiography (TTE) and BNP screening in patients at risk for cancer therapy-related cardiac dysfunction (CTRCD), performing ECG in those at risk for QTc prolongation, and extending thromboprophylaxis for 4–5 weeks after major surgery. Multidisciplinary collaboration remains essential. Preoperative evaluation should integrate patient- and procedure-related risks, avoiding unnecessary testing in low-risk surgeries or patients with good functional capacity. Functional capacity assessment using METs or validated tools such as the Duke Activity Status Index (DASI), ECG, biomarkers, and surgical risk calculators (e.g., RCRI, ACS NSQIP) guide decision-making. Coronary revascularization should follow standard indications, such as significant left main or multivessel disease, rather than routine prophylactic intervention, as demonstrated by trials like CARP and reinforced by registry data in high-risk anatomy. Antithrombotic management, including continuation of aspirin, selective use of dual antiplatelet therapy, and bridging strategies with cangrelor or heparin in high-risk patients, further influences perioperative outcomes. By combining guideline-directed care with oncology-specific considerations, clinicians can optimize surgical safety and reduce cardiovascular complications.

## 1 Introduction

Globally, more than 300 million surgical procedures are performed annually, with approximately 35 million occurring in the United States. Among these patients, 16%–47% undergo a cardiology consultation prior to non-cardiac surgery, reflecting the growing prevalence of cardiovascular risk factors in surgical populations, as well as the increasing awareness among clinicians of the benefit of pre-emptive intervention to mitigate cardiac adverse events. Data indicate that 45% of surgical patients over the age of 45 present with multiple cardiac risk factors, and nearly 25% have established atherosclerotic cardiovascular disease (ASCVD). Surgical intervention itself carries a significant risk, with an estimated 3% incidence of perioperative death, myocardial infarction, or ischemic stroke—corresponding to more than 150,000 perioperative cardiovascular events annually in the United States (1). Many of these adverse outcomes, including myocardial infarction, stent thrombosis, acute heart failure, hemodynamically significant arrhythmias, pulmonary embolism, bleeding complications, ischemic stroke, and death, are potentially preventable with appropriate perioperative management. In this context, a structured, evidence-based approach to perioperative cardiovascular evaluation is critical. This lecture (do you want this paper to be a review of a lecture of a broader review? Consider This paper will….) will synthesize current guidelines and literature on assessing and managing cardiovascular risk in patients undergoing non-cardiac surgery. We will discuss key objectives, risk calculation methods, strategies for risk evaluation, the impact of specific cardiovascular conditions on surgical outcomes, and therapeutic options, including pharmacologic and interventional approaches (2).

Within this broader context, patients with cancer undergoing surgery represent a uniquely high-risk group. Beyond traditional risk factors, tumor biology and prior cancer therapies may influence outcomes through effects on cardiovascular, pulmonary, hematologic, metabolic, nutritional, and immunologic systems. However, strong evidence-based guidelines directed at the cancer-patient population are lacking for many of these complex and overlapping scenarios, and clear consensus does not exist for several conditions discussed in this review. Accordingly, this article does not aim to provide definitive recommendations but rather to highlight key considerations and critical checkpoints that should contribute to pre-operative evaluation, particularly in patients requiring additional caution. Operative decision-making should therefore adopt a holistic, individualized approach that integrates patient-, disease-, and treatment-related factors. Looking forward, emerging approaches such as artificial intelligence may help integrate complex clinical variables, enhance risk stratification, and reduce perioperative misjudgment in this challenging population.

## 2 Risk evaluation

When assessing a cancer patient's perioperative risk, and the risk-benefit calculus, two primary components must be considered: resectability, i.e., the ability to successfully remove tumor or tumor burden. That component is generally addressed by the surgical-oncology team who, sometimes in broad discussion balance potential success with a risk. The second component, operability, addresses the risks of surgery for the specific patient, and encompasses cardiac, metabolic, pulmonary, nutritional and other potential parameters where abnormalities contribute to morbidity and mortality. The peri-operative clinician's overarching goal is to address, quantitate, and ultimately minimize these risks as effectively as possible to ensure optimal patient outcomes (3).

### 2.1 Evaluating surgical risk in non-cardiac surgery

According to the 2024 ACC/AHA guidelines, surgical procedures are stratified into three categories based on the estimated 30-day risk of major adverse cardiac events (MACE): low risk (<1%), intermediate risk (1%–5%), and high risk (>5%) (Table 1) (3).

**Table 1:** Surgical risk categories based on estimated 30-day cardiac risk, with representative procedure examples.

Risk Category	Estimated 30-Day Cardiac Risk	Examples
Low	<1%	Cataract surgery, superficial procedures
Intermediate	1%–5%	Orthopedic, urologic, intraperitoneal surgeries
High	>5%	Major vascular, thoracic, or intra-abdominal surgeries

In addition to procedural risk, several modifiers influence overall perioperative risk, including surgery duration, type of anesthesia, timing, and urgency. Urgency is further classified as emergent, urgent, time-sensitive, and elective (Table 2) (3). These factors collectively guide clinicians in tailoring perioperative cardiovascular evaluation and management strategies.

**Table 2:** Surgical urgency levels, definitions, and corresponding optimization potential before intervention.

Urgency Level	Definition	Optimization Potential
Emergent	Immediate threat to life/limb (<2 h.)	Minimal
Urgent	Required within 2–24 h	Limited
Time-sensitive	Ideally within weeks to 3 months	Moderate
Elective	Can be delayed indefinitely	Full

### 2.2 Patient-related risks

In addressing, patient-related risks, they can be identified and, in many cases, optimized before surgery. The cardiac evaluation begins with a thorough cardiovascular history and physical examination, as emphasized by contemporary guidelines, and extends, in instances where the patient is able to participate, to objective measures of functional capacity, such as metabolic equivalents (METs) or, in the altenative, validated tools like the Duke Activity Status Index (DASI). Additional layers of assessment—including electrocardiography, biomarker surveillance, and structured risk calculators—provide incremental precision in estimating perioperative outcomes. Integrating these elements within guideline-directed algorithms enables clinicians to stratify risk accurately, guide further testing when necessary, and implement targeted interventions. This approach is particularly critical in high-risk populations such as those with cancer, where baseline cardiovascular vulnerability and prior exposure to cardiotoxic therapies may augment perioperative risk.

#### 2.2.1 Functional capacity

Functional capacity, measured in metabolic equivalents (METs), is a key determinant of postoperative cardiovascular risk. A threshold of greater than or equal to 4 METs typically denotes adequate capacity, while the inability to climb two flights of stairs nearly doubles the risk of cardiovascular risk (9.6% vs. 5.2%) (4). Self-reported metrics offer important but limited insight. (Structured assessments add predictive value, especially in high-risk individuals, leading to a Class 2a recommendation for their use.).

A Class 2a, Level of Evidence B-NR recommendation from the *2024 AHA/ACC perioperative cardiovascular guideline* indicates that it is reasonable and generally advisable to use a structured assessment of functional capacity in patients undergoing elevated-risk non-cardiac surgery, (NCS). A Class 2a designation means the intervention is typically recommended because its benefits outweigh its risks, though it is not mandatory. The accompanying B-NR evidence level described in the *2024 AHA/ACC perioperative cardiovascular guideline* signifies that this guidance is supported by moderate-quality data from nonrandomized human studies**,** such as observational cohorts and registry analyses, which offer credible real-world evidence for clinical decision-making.

Based on this recommendation, patients facing elevated-risk NCS should undergo a structured evaluation of functional capacity, using validated instruments such as the Duke Activity Status Index (DASI)**.** Functional capacity is a strong predictor of perioperative cardiovascular events, and standardized tools provide more reliable risk estimation than informal clinical assessment. Incorporating these measures helps clinicians better stratify perioperative risk and determine whether additional cardiac testing or optimization is appropriate (5).

#### 2.2.2 Duke activity status score

The Duke Activity Status Score (DASI) quantifies functional capacity through a 12-item questionnaire, offering a semi-quantitative score that correlates with peak oxygen consumption (VO2). A DASI score <34 is associated with increased mortality and myocardial infarction risk, providing more precision than the subjective MET estimation (6). While the DASI is useful, potentially higher risks associated with surgical interventions in the cancer patient has not yet been quantitated.

#### 2.2.3 ECG and biomarker surveillance

Routine 12-lead ECG may be useful in patients with known coronary artery disease (CAD), structural heart disease, arrhythmias, or new cardiovascular symptoms. The 2024 guidelines give a Class 2a rating for ECG use in patients with cardiovascular problems, and a Class 2b for asymptomatic patients undergoing elevated-risk procedures. Class 2a suggests an intervention is reasonable and generally recommended because its benefits clearly outweigh its risks; class 2b suggests an intervention may be considered, but its benefit is less certain and supported by weaker or more limited evidence (5). Biomarker Assessment also enhances preoperative stratification. BNP/NT-proBNP levels are strongly predictive of perioperative cardiac events. Troponin testing may also be reasonable in these same populations that have a higher risk of cardiovascular complications (7, 8). Notwithstanding these hugely important generalizations, it must be noted that alterations in renal function and potentially other metabolic derangements may be reflected in the reports for biomarkers. This is an especially important consideration when these parameters are in the borderline range. Biomarker testing should be done so as to allow sufficient time for further evaluation an intervention.

#### 2.2.4 ACC/AHA algorithm

**Figure 1:**
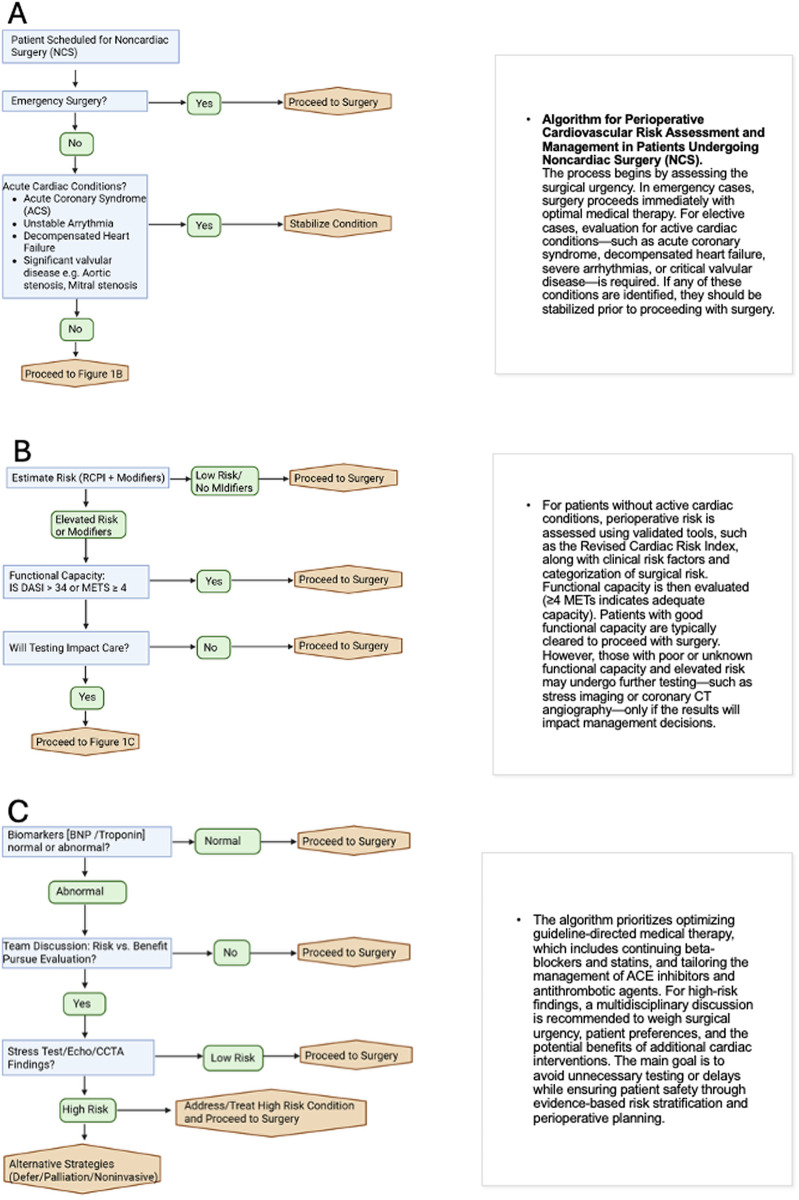
Algorithm for perioperative cardiovascular risk assessment in patients undergoing noncardiac surgery (AHA/ACC algorithm), (5). (A) Step 1: Determine Surgical Urgency. Start by assessing whether the surgery is emergency or elective. Emergency cases proceed directly to surgery with optimal medical therapy. Elective cases require screening for active cardiac conditions such as acute coronary syndrome, decompensated heart failure, severe arrhythmias, or critical valvular disease. If any are present, stabilize the patient before surgery. (B) Step 2: Assess Risk and Functional Capacity. For patients without active cardiac conditions, estimate perioperative risk using tools like the Revised Cardiac Risk Index and surgical risk categories. Evaluate functional capacity: ≥4 METs is considered adequate. Patients with good capacity usually proceed to surgery. Those with poor or unknown capacity and elevated risk may need further testing—such as stress imaging or coronary CT—only if results will change management. **(C)** Step 3: Optimize Therapy and Plan. Continue guideline-directed medical therapy, including beta-blockers and statins, and manage ACE inhibitors and antithrombotic agents individually. For high-risk findings, involve a multidisciplinary team to weigh surgical urgency, patient preferences, and benefits of intervention. The goal is to avoid unnecessary tests or delays while ensuring safety through evidence-based risk stratification and perioperative planning. Figure created with assistance from BioRender.com.

The 2024 ACC/AHA pathway combines clinical, functional, and biomarker data into a structured algorithm for perioperative cardiovascular risk assessment (5). The algorithm begins with CVD symptom evaluation and progresses through risk scoring, functional assessment (MET or DASI), and consideration of ECG/biomarkers. If uncertainty persists or abnormal findings arise, additional cardiac testing, such as ECHO, stress imaging, may be needed. This strategy is especially important in cancer patients whose baseline cardiovascular risk is often elevated due to prior exposure to cardiotoxic agents. The algorithm facilitates collaborative, multi-disciplinary decision making, and risk stratification (5) (Figure 1).

Although these algorithms are primarily adapted from established perioperative cardiovascular guidelines for the general population, a validated cancer-specific perioperative risk assessment algorithm has not yet been established. Given the heterogeneity of cancer types, treatment modalities, and patient comorbidities, current evidence remains insufficient to support the development of a robust oncology-specific algorithm, and individualized clinical judgment with multidisciplinary input remains essential. Future approaches, including artificial intelligence–based models integrating oncologic and cardiovascular variables, may help enable more precise cancer-specific perioperative risk stratification as additional evidence emerges.

### 2.3 Risk calculators

A comprehensive history and clinical evaluation are very important to perioperative cardiac risk stratification for patients undergoing non-cardiac surgery (NCS), especially in the oncology setting. The 2022 ESC guidelines recommend obtaining a detailed cardiovascular history and performing an accurate physical examination in all patients scheduled for NCS (3). The timing of pre-operative risk assessment ideally should be performed close enough to the anticipated surgery so as to minimize intercurrent events yet at a time that allows intervention as may be appropriate to optimize outcome and minimize risk (3)..

Validated perioperative risk assessment tools provide objective measures of cardiovascular risk and should be integrated into the decision-making process. Tools such as the Revised Cardiac Risk Index (RCRI), ACS NSQIP, and Surgical Outcome Risk Tool (SORT) have demonstrated reliability in predicting postoperative cardiac complications. Although the 2024 guidelines do not endorse a specific tool, they give a Class 2a recommendation for their use in patients known or suspected to have CVD undergoing NCS (5). Recent data suggests these tools remain valid in the cancer population, although further calibration for oncology-specific risk profiles may be warranted (9).

## 3 Coronary artery disease in preoperative evaluation

Coronary artery disease (CAD) is one of the most prevalent comorbidities encountered in surgical candidates. CAD affects approximately 18% of patients undergoing non-cardiac surgery, representing a substantial contributor to perioperative cardiovascular risk (10–12). CAD affects approximately 18% of patients undergoing non-cardiac surgery, representing a substantial contributor to perioperative cardiovascular risk (12). Surgical stress may precipitate myocardial ischemia through several mechanisms, including catecholamine-mediated plaque rupture leading to type 1 myocardial infarction, hemodynamic instability resulting in supply, demand mismatch, and thrombosis secondary to perioperative withdrawal of antiplatelet or anticoagulation therapy (13). A history of myocardial infarction increases perioperative major adverse cardiovascular events (MACE) by approximately 3.5-fold, while the presence of coronary stents, even beyond two years after placement, nearly doubles MACE risk (14). These observations underscore the importance of stratifying patients with known CAD using evidence-based, guideline-directed frameworks; these considerations may be of especial relevance in the cancer population due to under-appreciated surgical risk and the need for extensive surgical intervention.

CAD is more prevalent among patients with cancer, and this association is only partly explained by shared traditional risk factors such as smoking, hypertension, and diabetes (15, 16). Cancer itself promotes systemic inflammation, oxidative stress, and endothelial dysfunction, all of which accelerate atherosclerosis. In addition, certain cancer therapies—including chemotherapy, targeted agents, and radiation—can induce vascular injury and metabolic changes that further increase cardiovascular risk (17). These mechanisms underscore the importance of thorough cardiovascular assessment in cancer patients undergoing surgery, as their risk profile may differ significantly from non-cancer populations.

### 3.1 Stress testing

Both the ACC/AHA and ESC guidelines agree that routine stress testing is not recommended for preoperative evaluation in unselected patients. The ESC takes a stronger stance, recommending stress imaging in individuals with poor (<4 METs) functional capacity undergoing high-risk surgery, or in patients with known or highly probable CAD (14). Stress imaging has a high negative predictive value for perioperative MI and death, a finding supported by multiple prospective studies (18). However, its positive predictive value is limited, and only high-risk findings, such as moderate-to-large perfusion defects or extensive inducible ischemia, have consistent prognostic significance (13). This limitation explains why ACC/AHA guidelines offer only a Class IIb recommendation for stress testing in patients with elevated risk and unknown functional capacity. In the cancer population, co-morbidities are more commen and may influence the ultimate decision regarding stress testing.

### 3.2 Coronary CT angiography (CCTA)

The role of coronary CT angiography (CCTA) in ischemic evaluation is rapidly growing. Evidence demonstrates that CCTA adds incremental prognostic value beyond clinical risk scores such as the Revised Cardiac Risk Index, primarily due to its high negative predictive value (19). However, like stress testing, its positive predictive value remains modest. Several studies show that CCTA can overestimate perioperative cardiac risk by up to five-fold, with only partial improvement when combined with functional imaging (20, 21). Even in combined modalities, positive predictive value remains approximately 50%, limiting its usefulness as a standalone tool (22). Guidelines from both ACC/AHA and ESC therefore classify CCTA as a reasonable option in patients with poor functional capacity who are not candidates for standard stress testing.

However, in elderly patients or those with multiple atherosclerotic risk factors, extensive coronary artery calcification may limit the diagnostic accuracy of CCTA by impairing reliable evaluation of coronary lumen and flow. Consequently, the use of CCTA in asymptomatic patients should be individualized, and perioperative risk assessment should incorporate clinical risk stratification, functional capacity evaluation, biomarker data, and multidisciplinary clinical judgment when imaging findings are uncertain.

### 3.3 Invasive coronary angiography and coronary revascularization

**a)** Both the ESC and ACC/AHA guidelines concur that invasive coronary angiography should not be performed routinely for preoperative risk assessment (3, 5). Indications for angiography in the perioperative setting are identical to those in non-operative care and include suspected acute coronary syndrome, unstable or progressive angina, or high-risk ischemic features that warrant intervention regardless of surgical timing. In stable patients, routine preoperative catheterization has not demonstrated benefit and is therefore not recommended.

Similarly, the principles guiding coronary revascularization before non-cardiac surgery mirror those applied in general cardiology practice. Revascularization is appropriate for patients with acute coronary syndrome, significant left main coronary artery disease, or extensive inducible ischemia. Conversely, prophylactic or routine revascularization in stable individuals does not improve perioperative outcomes and should generally be avoided. These recommendations underscore the importance of individualized decision-making based on established clinical indications rather than surgical context alone.

Coronary revascularization prior to NCS was evaluated in the CARP trial, a randomized study of 510 patients with stable (CAD) undergoing major vascular surgery. The trial demonstrated that routine coronary revascularization did not reduce the incidence of 30-day myocardial infarction or long-term mortality over a median follow-up of 2.7 years (23). Importantly, patients with significant left main disease were excluded, and only a small proportion had triple-vessel disease, resulting in a cohort with moderate rather than extreme coronary risk. These findings established that prophylactic revascularization in stable patients offers no perioperative advantage and should not be performed solely to reduce surgical risk.

In contrast, a large registry of nearly 10,000 patients with high-risk coronary anatomy, (defined as ≥70% stenosis in all three epicardial vessels or ≥50% stenosis of the left main coronary artery) favored revascularization. In this population, revascularization was associated with a marked reduction in all-cause mortality and myocardial infarction, with survival curves clearly favoring intervention. These observations are consistent with autopsy studies showing that more than two-thirds of fatal perioperative myocardial infarctions occur in patients with left main or severe multivessel CAD (24). This underscores the prognostic significance of identifying high-risk anatomy before surgery.

Further supporting this concept, Bainey et al. conducted a landmark analysis of 9,016 patients with stable ischemic heart disease and high-risk coronary anatomy. Using inverse probability weighting, the study demonstrated that revascularization—whether by percutaneous coronary intervention (PCI) or coronary artery bypass grafting (CABG)—significantly reduced the composite endpoint of death or myocardial infarction (IPW-HR 0.62; 95% CI 0.58–0.66) (24). These findings reinforce the importance of distinguishing between moderate-risk and high-risk coronary disease in the preoperative setting, as the benefit of revascularization is confined to patients with severe anatomical disease rather than those with stable, lower-risk CAD.

### 3.4 Surgical timing after PCI

**Figure 2:**
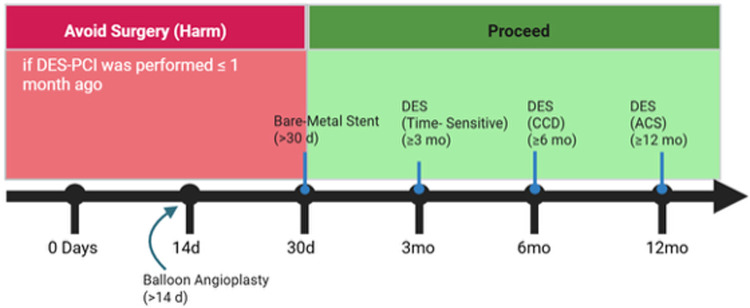
Optimal timing of noncardiac surgery after PCI (5). Recommended delays for noncardiac surgery after PCI: >14 days after balloon angioplasty, >30 days after bare-metal stent, ≥12 months after drug-eluting stent for ACS (≥3 months if time-sensitive), and ≥6 months for CCD (≥3 months if time-sensitive); avoid surgery within 1 month after DES-PCI due to harm. Figure created with assistance from BioRender.com.

Percutaneous coronary intervention is the conglomerate of widely used cardio-intervention techniques such as stents and angioplasty. Depending on the type of PCI done, the NCS timing after a patient has received PCI changes. AHA recommends that patients post-balloon angioplasty only (no stent) wait at least 14 days before undergoing NCS while those that receive BMS take 30 days or more before NCS (5). For DES, the timing varies depending on the cardiac condition. For example, patients post DES-PCI due to acute coronary syndromes (ACS) should wait at least 12 months before NCS (class I recommendation). Within this same patient group, if time sensitive NCS is indicated, patients should wait at least 3 months (class IIb recommendation) or wait at least 1 month as per situation (class III recommendation). On the other hand, patients who underwent DES-PCI for chronic coronary disease (CCD), should delay NCS for at least 6 months, 3 months for time sensitive NCS or 1 month as the situation and indications dictate, recommended as class IIa, IIb and III, respectively (5) (Figure 2).

To further emphasize the importance of timing of NCS post-PCI, a recent study by Cruden et al., reviewed the outcomes of non-cardiac surgery after PCI. This retrospective cohort study of 1,953 patients with 1 or more coronary stents placed prior to NCS found that perioperative major adverse cardiac events (MACE) were significantly higher within the first 42-day period post-stent placement (42.4% vs. 12.8%). This risk was even higher if the stent was placed for ACS within the first 42-day period (65% vs. 32%). Regardless, however, there remains a plateau of elevated risk for MACE in this patient group up to a year following stent placement (25).

In cancer survivors who have undergone percutaneous coronary intervention (PCI), the decision between drug-eluting stents (DES) and bare-metal stents (BMS) remains clinically significant. This is because anticipated oncologic therapies, the need for surgery, and the elevated bleeding risk associated with dual antiplatelet therapy (DAPT) strongly influence stent selection and DAPT duration. Although BMS were historically favored in patients with high bleeding risk or need for early surgery, recent evidence indicates that newer-generation DES, when paired with abbreviated DAPT, offer comparable safety and efficacy outcomes without increasing bleeding or mortality risk (26, 27).

## 4 Special considerations in preoperative cardiac assessment for cancer patients

The complexity of cancer patients undergoing surgery, characterized by the condition of the malignancy itself and the cardiotoxic effects of oncologic therapies forces physicians to move beyond routine protocols and adopt a more individualized approach.

1. **Heart Failure:** Heart failure is a key driver of risk: patients with heart failure have roughly three times the odds of postoperative mortality compared with those who have coronary-artery disease, and the danger rises further with decreasing left-ventricular ejection fraction (LVEF), hemodynamic decompensation, and worsening symptoms. The 2024 AHA/ACC/ACS guideline therefore advises targeted evaluation of LV function only in the presence of new or worsening symptoms rather than routine screening of stable, asymptomatic individuals, and emphasizes optimization of guideline-directed medical therapy (GDMT), volume status, and hemodynamics, while recommending a 3- to 4-day pause of sodium/glucose cotransporter 2 (SGLT2) inhibitors before surgery to avoid metabolic acidosis (5)(AHA/ACC/ACS et al., 2024).
2. **Aortic stenosis (AS):** Severe AS represents yet another critical obstacle. Aortic stenosis creates a fixed outflow obstruction that can precipitate peri-operative myocardial infarction or acute heart failure. The 2022 ESC guideline recommends valve replacement before any elective intermediate- or high-risk non-cardiac surgery in symptomatic patients, and real-world data confirm the benefit: among 491 patients with severe AS undergoing elective high-risk surgery, the 30-day major adverse cardiac event (MACE) rate was 5.4% in those who had prior aortic-valve replacement vs. 20.5% in those without, with symptomatic patients experiencing an even higher 35% MACE rate when left untreated (3, 28).
3. **Pericarditis:** Acute pericarditis or impending tamponade are clear contraindications to elective surgery and as such, require postponement until the emergency resolves. Nevertheless, patients with small-to-moderate, stable effusions may proceed, where preload optimization and the coordinated use of anti-inflammatory agents such as colchicine or immunosuppressants are a key clinical priority, which may require completion before surgery to minimize peri-operative complications (3).
4. **Atrial fibrillation (AF):** Pre-operative AF indicates underlying structural disease and thus predicts higher rates of mortality, heart-failure exacerbation, and stroke, making it another complex feature within the management of cancer patients. Management should begin by correcting reversible factors (e.g., anemia, infection), achieving rate control with a target heart rate below 110 bpm, and weighing the risks and benefits of peri-operative anticoagulation. Particularly, guidelines emphasize the need for strict outpatient follow-up in cases of new-onset perioperative atrial fibrillation, given its significant recurrence risk and the decision on whether long-term anticoagulation after surgery is needed (5). Because conventional risk calculators often underestimate the added burden of cardiotoxic cancer therapies, a lower threshold for additional testing, such as echocardiography or cardiac biomarkers, is warranted. The safest strategy is based on early, coordinated planning among several medical specialties such as cardiology, oncology, and surgical teams, ensuring that both cancer management and cardiac optimization are individualized and coordinated throughout the peri-operative period.
5. **Hypertension:** Hypertension is a major preoperative risk factor that causes a wide variety of side effects and requires careful evaluation and control. The 2024 American Heart Association (AHA) guidelines recommend continuing medical therapy for hypertension throughout the perioperative period if the patient's blood pressure remains below 180/110 mmHg (class IIa recommendation). Once the patient's systolic blood pressure exceeds 180 and/or diastolic blood pressure exceeds the 110 thresholds, AHA recommends deferring surgery if feasible (class IIb recommendation) (5). However, in terms of negative outcomes associated with hypertension, recent studies illustrate conflicting views. For example, Venkatesan et al. found that an even lower diastolic blood pressure threshold, specifically greater than or equal to 90, was associated with worse outcomes and increased 30-day mortality in patients (29). Another retrospective study that sampled around 58,000 patients by Walco et al., found that a systolic blood pressure greater than 160 was associated with worse outcomes only in patients with at least one revised cardiac risk index (RCRI) component while patients without any RCRI component (= 0) had no risk elevation for blood pressures up to 180/110 mmHg (30). It is important to note that patient blood pressure references for preoperative assessment should be based on baseline ambulatory blood pressure measurements (whenever possible) rather than that of the day of surgery to minimize the risk of white coat hypertension.

## 5 Antiplatelet and anticoagulant management: balancing thrombosis and bleeding risk in non-cardiac surgery

Managing antiplatelet and anticoagulant therapy in the perioperative setting is essential to balance thrombotic and bleeding risks, particularly in patients with prior PCI or high cardiovascular risk. Current AHA and ESC guidelines provide clear recommendations on continuing aspirin, timing of drug interruption, and when dual antiplatelet therapy (DAPT) should be maintained. Bridging strategies with intravenous agents such as cangrelor may be considered for patients at very high thrombotic risk, while oral anticoagulant management requires careful planning to avoid unnecessary bleeding. Special considerations, including thrombocytopenia in oncology patients, further complicate decision-making. This section reviews evidence-based approaches and guideline-directed strategies for optimizing antithrombotic therapy before non-cardiac surgery.

### 5.1 Antiplatelet therapy

Antiplatelet agents are an important perioperative medications that prevents negative outcomes such as thrombosis, especially in patients' post-percutaneous coronary interventions (PCI). The 2024 AHA guidelines have clear instructions for patients undergoing non-cardiac surgery (NCS) post-PCI. Class I recommendations include continuing aspirin (75–100 mg) in patients with prior PCI undergoing surgery whenever it is possible and substituting oral anticoagulant (OAC) monotherapy for aspirin during perioperative period until OAC can be reinitiated. Additionally, patients undergoing NCS with coronary artery disease (CAD) within 30 days of bare metal stent (BMS) placement or within 3 months of drug eluting stent (DES) placement should continue dual antiplatelet therapy (DAPT) unless the risk of bleeding outweighs the benefits for stent thrombosis prevention (5). The minimum time from drug interruption to restoration of platelet function or how long each antiplatelet therapy needs to be held pre-surgery varies depending on the type of drug used (3) The minimum time from drug interruption to restoration of platelet function varies by agent. For aspirin, platelet function typically returns after 4 days. Clopidogrel requires approximately 5–7 days (31), while prasugrel takes longer, about 7–10 days (32). Ticagrelor has the shortest recovery time among these, with platelet function restored in 3–5 days (33–35).

### 5.2 Bridging with cangrelor

The AHA 2024 guidelines recommend that perioperative bridging with IV antiplatelets such as cangrelor can be considered in patients at high thrombotic risk within 6 months of DES placement or within 30 days of BMS placement (5). The BRIDGE trial by Angiolillo and colleagues was a randomized, double blind, multicenter trial that studied 210 patients post ACS or stent placement waiting for coronary artery bypass graft surgery (CABG). The primary outcome evaluated was platelet inhibition in these patients given cangrelor or a placebo medication during perioperative period. The study found that cangrelor therapy pre-surgery increased platelet inhibition with no significant increase in major bleeding (36). The Monet Bridge trial is an on-going study evaluating bridging with cangrelor pre-NCS. The current MD Anderson protocol for cangrelor recommends using it in high-risk patients with recent stents, defined as st-elevation myocardial infarction (STEMI) within 6 months or non st-elevation myocardial infarction (NSTEMI)/unstable angina within 1 month.

### 5.3 Oral anticoagulants

For most patients undergoing NCS, OACs need to be held prior to the surgery, and OAC bridging is not routinely recommended since it increases bleeding risk. As a class IIa recommendation, the AHA states that only patients with high thrombotic risk (Table 3), undergoing vitamin K antagonist interruption, preoperative bridging with OAC, specifically parental heparin, can help reduce risk of thrombosis. After hemostasis is achieved, resuming OAC is warranted (5).

**Table 3:** Clinical conditions associated with high thrombotic risk in the perioperative setting.

High Thrombotic Risk Conditions
Mechanical MVR
Mechanical AVR + TE risk factor
Recent VTE
Valvular A-fib
A-fib + CHADS2-VASc > 6
A-fib + CHADS2-VASc 5–6 + recent stroke
Cardioembolic stroke < 3 months
Left ventricular thrombus < 3 months

### 5.4 Thrombocytopenia

10% of cancer patients present with thrombocytopenia (platelet count <100,000), putting this population at a higher risk of bleeding while not reducing thrombotic risk. There are no guidelines that suggest that these patients should receive stress testing or ischemic evaluation before undergoing surgery, however, surgical guidelines in general recommend having a platelet count >50,000 before procedures, and PCI guidelines establish that a count of 20,000–30,000 is sufficient for PCI. Therefore, patients being evaluated and considered for surgery can likely undergo coronary revascularization via stent placement beforehand if clinically indicated (37).

## 6 Other considerations: beta blockers

Early results that supported the benefit of beta-blockers prior to surgery have recently been called into question. The Perioperative Ischemic Evaluation (POISE) study was the first publication to show net harm caused by starting beta-blockade on the day of surgery. While these medications did improve cardiac outcomes and decreased myocardial oxygen demands, there was an overall increase in all-cause mortality caused by hypotension and stroke (38). Since then, the 2024 AHA/ACC/ACS/ASNC/HRS/SCA/SCCT/SCMR/SVM guidelines for perioperative cardiovascular management for noncardiac surgery recommend that patients on stable beta-blocker therapy can continue these medications, while recommending against starting beta-blockers within 7 days of surgery, as the risk of death outweighs the possible benefits (5).

## 7 Cancer-specific perioperative considerations

### 7.1 Introduction

Patients with cancer undergoing surgery represent a uniquely high-risk population. In addition to standard perioperative risk assessment, tumor-related factors and prior cancer therapies can significantly influence surgical outcomes across cardiovascular, pulmonary, hematologic, metabolic, nutritional, and immunologic systems. However, strong evidence-based guidelines are currently lacking for many of these complex and overlapping clinical scenarios, and no established consensus exists for several of the specific conditions discussed in the following sections. Therefore, this review does not aim to provide definitive recommendations but rather to highlight critical issues and key checkpoints that should be carefully considered during pre-operative evaluation, particularly in patients requiring heightened caution. Operative decision-making should take a holistic, individualized approach that accounts for the diverse and multifactorial challenges faced by each patient. Finally, emerging approaches such as artificial intelligence may offer future opportunities to integrate multiple clinical variables, support risk stratification, and reduce the likelihood of perioperative misjudgment in this complex patient population (39–42).

Patients with cancer undergoing surgery may have increased perioperative cardiovascular risk related to prior or ongoing oncologic therapies. The ESMO Clinical Practice Guidelines on cardiotoxicity management (43) and the 2022 ESC Cardio-Oncology Guidelines **(**44) highlight that agents such as anthracyclines, HER2-targeted therapies, fluoropyrimidines (e.g., 5-FU), platinum-based chemotherapy, and immune checkpoint inhibitors may contribute to cardiovascular complications including left ventricular dysfunction, ischemia, arrhythmias, and hypertension. Accordingly, perioperative cardiovascular evaluation should include careful review of prior cancer therapies, assessment of baseline cardiac function, and multidisciplinary collaboration between oncology and cardiology teams when treatment-related cardiotoxicity is suspected.

The American Society of Clinical Oncology (ASCO) guidelines further recommend baseline cardiovascular risk assessment and surveillance in patients receiving potentially cardiotoxic therapies, particularly anthracyclines and HER2-targeted agents (45). These strategies may include clinical evaluation, cardiac imaging, and biomarker monitoring in selected high-risk patients. In the perioperative setting, awareness of prior cardiotoxic therapy and available surveillance data may assist in identifying patients at increased risk of cardiovascular complications.

In addition, International Cardio-Oncology Society (ICOS) position statements emphasize standardized risk stratification and multidisciplinary collaboration to identify and manage treatment-related cardiovascular complications (46). Contemporary cardio-oncology frameworks also emphasize broader considerations captured in the TBIP framework—including thrombotic risk, bleeding risk, drug–drug interactions, and patient preferences—which are particularly relevant in patients undergoing cancer surgery. In this context**,** cancer-associated thrombosis (CAT) represents a major contributor to perioperative morbidity and mortality. Contemporary guidance from ASH, ESMO, and ASCO highlights that thrombotic risk in cancer is influenced by tumor type, stage, systemic therapy, immobility, central venous access, and surgical factors, while bleeding risk may vary depending on tumor location, thrombocytopenia, renal function, and concurrent therapies (47–49). These frameworks support individualized perioperative risk assessment that balances thrombosis and bleeding risks when considering perioperative anticoagulation strategies.

At the same time, recent cardio-oncology literature highlights persistent evidence gaps regarding optimal perioperative cardiovascular management in patients with cancer (50, 51). In some situations, recommendations from general perioperative cardiology guidelines and cardio-oncology frameworks may not fully align. Therefore, individualized clinical judgment and multidisciplinary discussion remain essential when applying these recommendations to perioperative decision-making in this complex patient population.

### 7.2 Tumor-related factors

#### 7.2.1 Disease burden and stage

1. **Assessment of Physiological Reserve:** Age alone does not adequately predict surgical risk in patients with advanced cancer. Preoperative frailty and malnutrition are strong predictors of postoperative complications and decreased survival in surgical oncology (52). Although several frailty tools exist, there is no single universally adopted standard for measuring physiological reserve in advanced cancer patients. Therefore, risk assessment should be individualized and holistic rather than relying on a single scoring system.
2. **Site Specific Metastatic Evaluation**: Metastatic disease significantly affects a patient's ability to tolerate surgery. Evaluation must go beyond simple organ clearance and instead consider how multiple metastatic sites interact with the physiological stress of surgery. Currently there is no unified preoperative framework that integrates multi-organ metastatic burden into surgical risk modeling.

Examples of organ-specific metastatic effects include:

• **Lung:** Impaired gas exchange and reduced baseline oxygenation. In advanced cases, pulmonary vascular resistance may increase.
• **Liver:** Reduced drug metabolism, hypoalbuminemia, and in cases of extensive involvement: coagulopathy.
• **Bone:** Increased risk of pathologic fractures, hypercalcemia, and intraoperative positioning challenges (e.g., nerve injury or fracture risk during prolonged positioning).
• **Brain:** Elevated intracranial pressure, altered mental status, and increased seizure risk, which complicate anesthetic management and postoperative neurological monitoring.
3. **Systemic Inflammatory Impact**: Many tumors induce a hypermetabolic and pro-inflammatory state. Elevated preoperative inflammatory markers have been associated with increased postoperative complications and worse outcomes in cancer patients (53). These findings suggest that systemic inflammation may reflect reduced physiological reserve and increased surgical vulnerability.

#### 7.2.2 Paraneoplastic syndromes

Endocrine and metabolic abnormalities may complicate perioperative management:

1. **SIADH:** SIADH is commonly associated with cancer, most often as a paraneoplastic syndrome, particularly in small cell lung carcinoma, where ectopic ADH secretion causes water retention and dilutional hyponatremia. It may also occur secondary to tumor effects, CNS or pulmonary involvement, or cancer therapies. In oncology patients, hyponatremia can worsen fatigue, cognition, and overall functional status, potentially reflecting active disease or progression. Perioperatively, recognizing SIADH is important because untreated hyponatremia increases neurologic and anesthetic risk; management focuses on correcting electrolyte abnormalities while addressing the underlying malignancy to improve perioperative safety (54, 55).
2. **Hypercalcemia of malignancy:** Hypercalcemia of malignancy is a common paraneoplastic disorder in advanced cancers, often caused by PTHrP secretion, osteolytic metastases, or increased vitamin D production. Elevated calcium can lead to dehydration, neurocognitive symptoms, muscle weakness, and cardiac conduction abnormalities, increasing perioperative risk. Pre-operative evaluation should include corrected or ionized calcium, renal function, hydration status, electrolyte assessment, and ECG monitoring. Management focuses on stabilizing calcium levels and correcting volume depletion, primarily with intravenous hydration, with bisphosphonates or calcitonin used as needed. Elective surgery should be delayed until metabolic abnormalities are controlled whenever possible (56, 57).
3. **Ectopic hormone production:** Ectopic hormone production occurs when tumors secrete hormones independent of normal endocrine control, causing syndromes such as ectopic ACTH (Cushing syndrome), hypoglycemia, or electrolyte disturbances that increase perioperative risk. Pre-operative evaluation should screen for endocrine dysfunction through clinical assessment and targeted laboratory testing, as these conditions may cause hypertension, hyperglycemia, muscle weakness, or volume imbalance. Management focuses on stabilizing metabolic and hormonal abnormalities, coordinating with endocrinology and oncology, and delaying elective surgery until adequate control is achieved whenever possible (58–60).

### 7.3 Cancer treatment–related toxicities

#### 7.3.1 Chemotherapy

Chemotherapeutic agents may cause persistent organ dysfunction even years after treatment.

##### 7.3.1.1 Cardiovascular

Anthracyclines (e.g., doxorubicin, daunorubicin) cause dose-dependent cardiotoxicity leading to dilated cardiomyopathy and heart failure, with risk increasing with cumulative dose, prior chest radiation, and underlying cardiovascular disease; pre-operative assessment should focus on prior exposure, heart failure symptoms, and echocardiographic evaluation, with optimization of cardiac function before surgery. HER2-targeted therapies (e.g., trastuzumab) cause typically reversible left ventricular dysfunction that is not dose-dependent and often improves after treatment interruption; evaluation relies on recent echocardiographic monitoring and assessment of functional status, with perioperative planning aimed at supporting reduced but potentially recoverable cardiac reserve (61, 62). Fluoropyrimidines (5-FU, capecitabine) most commonly cause coronary vasospasm resulting in transient ischemia rather than structural myocardial damage (63); pre-operative evaluation should specifically screen for chemotherapy-related chest pain, and management focuses on preventing ischemic triggers, maintaining stable hemodynamics, and using antianginal therapy when appropriate.

##### 7.3.1.2 Pulmonary

**Bleomycin:** Bleomycin is associated with interstitial lung disease and pulmonary fibrosis due to oxidative lung injury, resulting in reduced pulmonary reserve and increased oxygen sensitivity. Risk is higher with greater cumulative dose, older age, renal dysfunction, and prior thoracic radiation. Pre-operative evaluation should assess for dyspnea, dry cough, and reduced exercise tolerance, with chest imaging and pulmonary function testing (especially DLCO) considered to detect subclinical toxicity. Because pulmonary complications may occur long after treatment, prior bleomycin exposure should always be identified. Perioperative management focuses on minimizing oxygen-related lung injury by using the lowest effective FiO₂, careful fluid management, and cautious planning in patients with significant pulmonary impairment (64).

##### 7.3.1.3 Hematologic

Systemic anticancer therapy can suppress hematopoiesis, leading to neutropenia, anemia, and thrombocytopenia, all factors that alter perioperative infection risk, bleeding risk, transfusion requirements, and physiologic reserve. These effects are often temporally patterned relative to treatment cycles, making surgical timing and perioperative planning critical components of risk mitigation (65).

1. **Myelosuppression:** Treatment-related myelosuppression reflects decreased bone marrow production following systemic therapy, with severity influenced by regimen intensity, cumulative exposure, and baseline marrow reserve; because nadir and recovery periods are often predictable, perioperative decisions should interpret cytopenias in relation to recent therapy rather than isolated laboratory values. Pre-operative assessment should include review of recent treatment timing, anticipated nadir/recovery, prior severe cytopenias or febrile neutropenia, and a CBC with differential close to surgery, using trends to guide timing and perioperative precautions. When elective and oncologically feasible, surgery should be postponed during profound or worsening multilineage suppression to reduce infectious and bleeding complications (65).
2. **Neutropenia:** Neutropenia increases risk of bacterial and fungal infections and may mask typical inflammatory signs, complicating early detection of postoperative infection; perioperative data show that severe neutropenia is associated with higher postoperative morbidity and should be incorporated into operative risk and timing decisions (66). Pre-operatively, ANC should be interpreted in the context of treatment timing, with elective surgery often deferred when severe neutropenia is present and oncologically acceptable (66). For urgent procedures, heightened infection surveillance, appropriate antimicrobial strategies, and early oncology/hematology coordination are recommended, while growth factor use should follow oncology-driven indications consistent with ASCO guidance rather than routine perioperative use (65).
3. **Anemia:** Anemia in patients receiving systemic anticancer therapy is often multifactorial and is associated with increased perioperative morbidity and higher transfusion risk; management should focus on early identification and treatment of reversible causes, with transfusion guided by restrictive, symptom- and comorbidity-based strategies in stable patients (67, 68). Pre-operative evaluation should include early hemoglobin assessment to allow targeted treatment (e.g., iron replacement) when possible, use of evidence-based restrictive red blood cell transfusion thresholds with individualized adjustment for bleeding, ischemic heart disease, or symptoms, and consideration of erythropoiesis-stimulating agents only for oncology guideline–supported indications given thromboembolic and outcome risks (67–69).
4. **Thrombocytopenia:** Thrombocytopenia in cancer patients may result from reduced production, marrow infiltration, immune destruction, or consumption and increases perioperative bleeding risk while also reflecting overall disease severity; evidence supports prophylactic platelet transfusion in severe hypoproliferative thrombocytopenia, and preoperative thrombocytopenia is associated with worse surgical outcomes, supporting individualized planning based on procedure risk (70, 71). Pre-operative management should assess platelet trend and mechanism, define procedure-specific platelet targets, plan transfusion strategies for higher-risk surgeries, and consider hematology consultation for immune, refractory, or consumptive causes (70, 71).

##### 7.3.1.4 Renal

Many chemotherapeutic agents, particularly platinum-based drugs (cisplatin, carboplatin, oxaliplatin), can cause acute kidney injury (AKI) and contribute to chronic kidney disease, making renal assessment essential in pre-operative evaluation (72). Repeated exposure may lead to persistent GFR decline, especially when combined with dehydration or additional nephrotoxins such as ICIs, VEGF inhibitors, ifosfamide, high-dose methotrexate, NSAIDs, ACE inhibitors, or aminoglycosides, highlighting the importance of reviewing multidrug interactions (73). Pre-operative assessment should include detailed treatment history, cumulative dose, timing of therapy, and prior toxicity (74), along with evaluation of renal function using creatinine and estimated GFR, with cystatin C considered in patients with prior platinum exposure and reduced muscle mass; emerging biomarkers such as NGAL and KIM-1 may detect subclinical injury but are not yet standard (75–77). Electrolyte abnormalities should be corrected before surgery, and nephrotoxic drugs such as NSAIDs should be avoided to reduce perioperative renal risk (74).

#### 7.3.2 Radiation therapy

Radiation therapy can produce progressive, delayed tissue injury that may appear months to decades after treatment, making it an important consideration during pre-operative evaluation in cancer patients. The degree of injury depends on radiation field, cumulative dose, fractionation schedule, and concurrent chemotherapy (78). Radiation-related damage is often characterized by chronic inflammation, fibrosis, microvascular injury, and reduced tissue compliance, which may significantly alter physiologic reserve even in patients who appear clinically well. A detailed oncologic history, including treatment site and timing, is essential for identifying organ systems at risk.

Cardiac and pulmonary complications are particularly relevant when radiation involves the thorax or mediastinum. Cardiac effects may include accelerated coronary artery disease, valvular thickening or calcification, conduction abnormalities, pericardial disease, and restrictive cardiomyopathy (78, 79), all of which can increase perioperative cardiac risk. Pulmonary injury may manifest as interstitial fibrosis, decreased lung compliance, reduced diffusion capacity, and impaired gas exchange, leading to increased risk of postoperative respiratory failure (80). Pre-operative evaluation should therefore include assessment of exercise tolerance, cardiopulmonary symptoms, and targeted testing such as echocardiography, ECG, chest imaging, or pulmonary function testing when indicated.

Radiation also causes significant soft tissue and vascular changes that may influence surgical planning and wound healing. Fibrosis, tissue fragility, poor vascularity, and reduced regenerative capacity can increase the risk of difficult dissection, poor healing, infection, and postoperative complications, especially in previously irradiated fields. During pre-operative assessment, clinicians should review prior radiation maps and cumulative doses, examine tissue quality at the operative site, and anticipate airway or positioning challenges if head, neck, or thoracic regions were treated. Early recognition of these radiation-related effects enables multidisciplinary planning, optimization of organ function, and safer anesthetic management.

#### 7.3.3 Immunotherapy

Immune checkpoint inhibitors (ICIs) can cause immune-mediated myocarditis, a rare but potentially fatal complication that may present with arrhythmias, heart failure, chest pain, dyspnea, or nonspecific symptoms. Because onset may occur during or months after therapy, prior ICI exposure should be specifically reviewed during pre-operative assessment. Evaluation should include careful symptom review, ECG, cardiac biomarkers (e.g., troponin), and echocardiography when indicated, with cardiac MRI or cardiology consultation considered if myocarditis is suspected. Perioperative management focuses on early recognition, risk stratification, and coordination with oncology and cardiology teams, with stabilization and postponement of elective surgery when active myocarditis or significant cardiac dysfunction is present (62, 81).

### 7.4 Hematologic and thrombotic considerations

Cancer creates a hypercoagulable state, and thrombotic risk is further increased by systemic therapies, central venous catheters, immobility, and surgery. Chemotherapy agents strongly associated with venous thromboembolism (DVT/PE) include platinum-based drugs (especially cisplatin), antiangiogenic agents (e.g., bevacizumab), immunomodulatory drugs (thalidomide and lenalidomide), hormonal therapies, and some targeted therapies. During pre-operative evaluation, thrombotic risk assessment should include cancer type and stage, prior thrombosis, ongoing therapies, anticoagulation use, and symptoms suggestive of occult VTE (leg swelling, dyspnea, unexplained tachycardia). Management focuses on individualized perioperative thromboprophylaxis, balancing bleeding vs. thrombosis risk, and early recognition of perioperative thromboembolic events through coordinated surgical, anesthesia, and oncology planning (82–86).

Cytopenias (anemia, thrombocytopenia, and neutropenia) are common in cancer patients due to treatment or marrow involvement and significantly influence perioperative risk. Pre-operative assessment should evaluate severity and trends in blood counts, bleeding risk, and infection susceptibility, with optimization strategies such as transfusion, growth factor support, or delay of elective surgery when severe abnormalities are present. Multidisciplinary coordination and careful perioperative monitoring are essential to reduce bleeding, infection, and hemodynamic complications (85, 87–89).

### 7.5 Nutritional and functional status

#### 7.5.1 Malnutrition and cachexia

Malnutrition and cachexia are common in patients with malignancy, particularly those with gastrointestinal, head and neck, and advanced-stage cancers. Cancer-associated cachexia is characterized by systemic inflammation, loss of skeletal muscle mass, and metabolic alterations that cannot be fully reversed by simple nutritional supplementation. These changes reduce physiologic reserve and increase vulnerability to surgical stress, making nutritional assessment an essential component of pre-operative evaluation. During pre-operative assessment, clinicians should evaluate recent weight loss, body mass index, oral intake, muscle mass, and functional status. Laboratory markers such as albumin and prealbumin may provide supportive information but should be interpreted in the context of inflammation and overall clinical status. Malnutrition is strongly associated with poor wound healing, higher rates of postoperative infection, delayed recovery, and prolonged hospital stay, emphasizing the need to recognize high-risk patients early (90–93).

Pre-operative management focuses on nutritional optimization and multidisciplinary planning. When feasible, nutritional intervention—including oral supplementation, enteral feeding, or parenteral nutrition in selected cases—should be initiated prior to major surgery. Assessment of frailty and functional capacity may further guide perioperative planning and risk stratification. Early recognition and correction of malnutrition help improve surgical outcomes, reduce complications, and support recovery in cancer patients undergoing operative procedures (93–96).

#### 7.5.2 Functional capacity

Functional capacity is a key predictor of perioperative risk in cancer patients, as reduced physiologic reserve limits the ability to tolerate surgical stress and recover postoperatively. Decline in performance status, such as an ECOG score ≥2**,** is associated with increased postoperative complications, longer hospitalization, and higher mortality (ref). Functional impairment in cancer patients may result from tumor burden, treatment-related toxicity, malnutrition, or comorbid disease, making routine assessment essential during pre-operative evaluation. During pre-operative assessment, clinicians should evaluate baseline activity level, exercise tolerance, mobility, and independence in activities of daily living. Objective assessment tools—such as functional performance scales, gait speed, stair-climbing ability, or cardiopulmonary exercise testing when available—help estimate functional reserve more reliably than subjective assessment alone. Identifying reduced functional capacity allows for better risk stratification and informs decisions regarding surgical candidacy and anesthetic planning (94, 97–99).

Pre-operative management should focus on optimizing functional status whenever possible through multidisciplinary interventions, including physical therapy, nutritional support, and management of reversible medical conditions. Prehabilitation programs may improve endurance and postoperative recovery in selected patients. Incorporating functional capacity into perioperative planning helps guide the intensity of monitoring, postoperative care needs, and overall risk–benefit discussions, ultimately improving outcomes in cancer patients undergoing surgery (100–103).

### 7.6 Cancer type–specific considerations

#### 7.6.1 Head and neck cancer

Head and neck cancer poses significant perioperative airway challenges due to tumor burden, prior surgery, and radiation-induced fibrosis, which may distort anatomy and increase the risk of difficult ventilation or intubation. Pre-operative evaluation should include careful airway assessment (mouth opening, trismus, neck mobility, and oropharyngeal visualization) and review of symptoms such as stridor, dyspnea, dysphagia, or voice changes, with imaging or endoscopic evaluation considered when indicated. Pre-operative planning should include a clear airway strategy with backup options, consideration of awake or advanced airway techniques in high-risk patients, and close coordination among anesthesia, surgical, and oncology teams to improve perioperative safety (104–106).

#### 7.6.2 Lung cancer

Lung cancer patients often have reduced baseline pulmonary reserve, and surgical resection further decreases functional capacity; therefore, pre-operative assessment of FEV1 and DLCO is essential, as a DLCO <40% predicted is associated with increased risk of postoperative respiratory failure (107). Pulmonary resection can also acutely increase pulmonary vascular resistance, leading to right ventricular overload, dysfunction, and arrhythmias, particularly after larger resections such as pneumonectomy (108). Although pulmonary hypertension was historically a contraindication to major lung surgery, selected patients with mild-to-moderate disease may undergo resection following individualized, holistic risk assessment due to the absence of definitive thresholds (109). Additionally, thoracic surgery disrupts chest wall mechanics and effective coughing, with postoperative pain increasing risk of atelectasis and pulmonary complications; therefore, assessment of ability to participate in pulmonary rehabilitation is important to support recovery (110).

#### 7.6.3 Gastrointestinal malignancies

Gastrointestinal malignancies create important perioperative challenges due to altered anatomy, impaired nutrition, and organ dysfunction, requiring careful assessment of disease burden, prior therapy, and metabolic status. Aspiration risk is especially high in esophageal cancer because of obstruction, delayed gastric emptying, or impaired motility; evaluation should include symptoms such as dysphagia, regurgitation, reflux, and weight loss. Liver involvement or underlying cirrhosis may alter drug metabolism, coagulation, and fluid balance, necessitating assessment of liver function and signs of portal hypertension. Because malnutrition is common and associated with poor healing and infection risk, pre-operative evaluation should include nutritional and functional assessment, with anesthetic planning accounting for aspiration precautions, hepatic dysfunction, and reduced physiologic reserve through multidisciplinary coordination (93, 111).

#### 7.6.4 Hematologic malignancies

Hematologic malignancies confer perioperative risk that is frequently underestimated by conventional surgical risk tools because risk is driven not only by measured cytopenias but also by qualitative immune dysfunction (e.g., hypogammaglobulinemia, impaired cellular immunity), therapy-related immune suppression (e.g., anti-CD20, cellular therapies), and organ involvement that alters cardiopulmonary reserve and hemodynamic tolerance (112, 113).

##### 7.6.4.1 Profound immunosuppression

Immunosuppression in hematologic malignancies is often multifactorial and may persist despite normal neutrophil counts due to humoral and cellular immune dysfunction, increasing susceptibility to severe or atypical infections and complicating perioperative detection (112). Cellular therapies such as CAR-T and high-dose chemotherapy or stem cell transplantation add prolonged cytopenias, delayed immune recovery, and sustained infection risk, requiring structured screening, prophylaxis, and early infectious disease involvement, often extending months beyond treatment (113–116). Pre-operative assessment should therefore go beyond CBC to include infection history, recent immunosuppressive therapies, and prophylaxis status, with perioperative antimicrobial strategies aligned with hematology/oncology and transplant-based guidance according to the patient's stage of immune reconstitution (112, 113, 115, 116).

##### 7.6.4.2 Infiltrative cardiomyopathy (e.g., amyloidosis in myeloma)

Plasma cell dyscrasias may be complicated by AL amyloidosis with cardiac involvement, causing restrictive cardiomyopathy, diastolic dysfunction, conduction abnormalities, autonomic instability, and marked preload/afterload sensitivity that increase risk of hypotension, arrhythmias, and perioperative decompensation (117, 118). Consensus recommendations support structured evaluation using clinical red flags, cardiac biomarkers, and multimodality imaging (echocardiography, cardiac MRI, and radionuclide imaging) to confirm diagnosis and guide risk stratification (119, 120), with NT-proBNP and troponin-based staging frameworks helping assess severity and prognosis (121). Pre-operative assessment should include ECG, echocardiography, and biomarker evaluation in suspected cases, management should follow restrictive cardiomyopathy principles with careful hemodynamic planning, and multidisciplinary coordination or specialist referral is recommended when diagnostic uncertainty remains (117–121).

#### 7.6.5 Endocrine tumors

Endocrine tumors pose distinct perioperative risks due to hormonally mediated cardiovascular and hemodynamic instability. In contrast to cytotoxic treatment-related toxicity, morbidity in these patients is primarily driven by bioactive hormone excess and its chronic cardiovascular sequelae. Preoperative identification and targeted physiologic optimization are therefore critical.

##### 7.6.5.1 Pheochromocytoma

Pheochromocytomas and paragangliomas (PPGLs) are catecholamine-secreting neuroendocrine tumors that cause significant blood pressure instability, arrhythmias, and end-organ effects, creating major perioperative challenges (122). Catecholamine-induced cardiomyopathy, including Takotsubo and dilated phenotypes, is increasingly recognized, and pre-operative optimization with α-blockade and heart failure therapy has been associated with recovery of ventricular function and acceptable intraoperative hemodynamic control (123). Pre-operative management typically includes 7–14 days of α-adrenergic blockade, with β-blockade added after adequate α-blockade for tachyarrhythmia control (122), while short-acting α1-antagonist infusions such as urapidil may reduce intraoperative hemodynamic volatility (124). Comprehensive cardiovascular assessment, including echocardiography, is recommended to identify cardiomyopathy, and intraoperative planning should anticipate severe hemodynamic fluctuations during induction and tumor manipulation, supported by invasive monitoring and multidisciplinary coordination among endocrinology, cardiology, anesthesia, and surgery teams (122, 124).

##### 7.6.5.2 Carcinoid tumors

Serotonin-secreting neuroendocrine tumors (NETs) may cause carcinoid syndrome and carcinoid heart disease (CHD), in which chronic exposure to vasoactive mediators leads to fibrotic degeneration of right-sided cardiac valves, tricuspid and pulmonary regurgitation, progressive right ventricular volume overload, and eventual right heart failure; cardiac involvement occurs in up to half of patients and is a major determinant of mortality (125, 126). Perioperative stress or tumor manipulation may trigger carcinoid crisis, causing severe hemodynamic instability due to massive mediator release (125). Pre-operative evaluation should include transthoracic echocardiography and NT-proBNP screening for cardiac involvement, while management includes hormonal control with somatostatin analogues and perioperative intravenous octreotide in at-risk patients, with careful assessment of right ventricular function and tailored fluid management in those with established CHD (125, 126).

### 7.7 Timing of surgery relative to cancer therapy

The timing of pre-operative cardiac evaluation requires integration of several crucial variables forming a continuum requiring careful consideration. A simple algorithm is probably not possible, but some basic thoughts may be helpful.

Patients with few underlying risk factors represent low surgical risk; the timing of a preoperative evaluation when required and when based on either the severity of the intervention to be performed, or simply the chronologic age of the patient, may be variable: too much time between the evaluation and the intervention could allow for intercurrent conditions to arise which could change overall risk. Delaying the evaluation in order to give the surgical team a timely and accurate assessment close to the anticipated time of the intervention does not allow for optimization of blood pressure or the control or elimination of an infection. Too brief an interval between the evaluation and scheduled surgery may dictate the need for surgical delay and rescheduling (127, 128).

As the degree of co-existing conditions and risk-factors increases, the timing becomes increasingly problematic and the factor related to the urgency of the intervention may become the limiting factor. Emergent surgical intervention, as may occur with ruptured viscus or uncontrolled internal bleeding, may have to forgo any pre-operative cardiac assessment other than to recommend post-surgical monitoring and control of altered hemodynamics.

In considering preoperative assessment from the cardio-oncology perspective some special examples come to mind. Recent chemotherapy may increase the risk of infection and bleeding and can be associated with delayed wound healing. When possible, surgery should be timed so that a sufficient degree of cellular and metabolic recovery has taken place to make the risks of these complications acceptable when balanced with the overall condition of the patient undergoing chemotherapy. The need to delay the subsequent cycle of chemotherapy must also be considered. The decision regarding timing should take the perspectives of the oncology and surgical teams into consideration; the cardio-oncologist's input in anticipating and managing cardiac sequelae should be an important component of the perioperative team as the risks and benefits are addressed (44).

Patients who have undergone stem cell transplantation present additional concerns as recovery is variable and often unpredictable. Transfusion of blood products including platelets provides varying degrees of stabilization, sometimes of limited duration. Availability of blood products and other supportive care modalities should be anticipated. Cardiac events may be increased as the stress these patients undergo evolves. The cardio-oncologist should be prepared to assess ischemic concerns, dysrhythmic events, and stress-related alterations, as well as pericardial and metabolic abnormalities that may arise more commonly in these vulnerable populations (45, 129, 130).

### 7.8 Role of artificial intelligence in perioperative evaluation

Artificial intelligence (AI) has emerging potential to improve perioperative evaluation by integrating large volumes of heterogeneous clinical data, including demographics, comorbidities, laboratory values, imaging findings, treatment history, and procedural risk factors. In complex populations such as patients with cancer, AI-based models may assist in identifying subtle risk patterns that are difficult to capture through traditional scoring systems alone, enabling more precise prediction of perioperative cardiovascular, thrombotic, and pulmonary complications. Machine learning approaches can support individualized risk stratification, early detection of clinical deterioration, and decision support for diagnostic testing or perioperative monitoring intensity. In addition, AI tools may help synthesize oncology-related variables—including prior cardiotoxic therapies, treatment timing, and systemic complications—into practical risk assessments that complement clinician judgment. However, current applications remain largely investigational, and limitations including data quality, model bias, interpretability, and generalizability must be addressed before widespread adoption. Rather than replacing clinical decision-making, AI is likely to serve as a supportive tool that enhances multidisciplinary perioperative planning by improving risk awareness and reducing the likelihood of misjudgment in complex, high-risk patients (42).

## 8 Conclusions

Pre-operative evaluation in cancer patients must extend beyond conventional surgical risk assessment. Tumor biology, prior cancer therapies, systemic complications, and functional status all contribute significantly to perioperative risk, and many oncologic surgeries are time-sensitive rather than truly elective, requiring careful weighing of risks and benefits. Patients with cancer may be at increased risk for a wide spectrum of cardiovascular complications, including coronary and peripheral arterial disease, cardiomyopathy, valvular dysfunction, arrhythmias, pericardial disease, bleeding, and thromboembolic events. Although no definitive guidelines exist to fully tailor pre-operative strategies in this population, several practical adjustments may help improve perioperative outcomes. These include incorporating oncologic history into risk stratification, lowering the threshold for transthoracic echocardiography and BNP screening in patients at risk for cancer therapy–related cardiac dysfunction, considering earlier pre-operative ECG evaluation in those at risk for QTc prolongation, and extending thromboprophylaxis for 4–5 weeks following major surgery to reduce thrombotic risk.

Pre-operative evaluations are a frequent component of cardiology consultation and should integrate both patient-related and procedure-related risks. In general, additional testing is not required for low-risk procedures, low-risk patients, or individuals with good functional capacity, and indications for further cardiac testing largely parallel those used in the general population. An important distinction applies to patients with stable chronic coronary disease, in whom revascularization should be considered when significant left main or major vessel disease is present. As emphasized throughout this review, however, patients with cancer represent a uniquely complex population requiring heightened clinical awareness and individualized planning. Close communication among oncology, anesthesia, cardiology, and surgical teams remains essential to optimize perioperative management and outcomes.
